# Multi-frequency steady-state visual evoked potential dataset

**DOI:** 10.1038/s41597-023-02841-5

**Published:** 2024-01-04

**Authors:** Jing Mu, Shuo Liu, Anthony N. Burkitt, David B. Grayden

**Affiliations:** 1 Department of Biomedical Engineering, The University of Melbourne, Parkville, Victoria 3010 Australia; 2 Graeme Clark Institute, The University of Melbourne, Parkville, Victoria 3010 Australia

**Keywords:** Biomedical engineering, Electroencephalography - EEG, Brain-machine interface

## Abstract

The Steady-State Visual Evoked Potential (SSVEP) is a widely used modality in Brain-Computer Interfaces (BCIs). Existing research has demonstrated the capabilities of SSVEP that use single frequencies for each target in various applications with relatively small numbers of commands required in the BCI. Multi-frequency SSVEP has been developed to extend the capability of single-frequency SSVEP to tasks that involve large numbers of commands. However, the development on multi-frequency SSVEP methodologies is falling behind compared to the number of studies with single-frequency SSVEP. This dataset was constructed to promote research in multi-frequency SSVEP by making SSVEP signals collected with different frequency stimulation settings publicly available. In this dataset, SSVEPs were collected from 35 participants using single-, dual-, and tri-frequency stimulation and with three different multi-frequency stimulation variants.

## Background & Summary

Brain-Computer Interfaces (BCIs), also called Brain-Machine Interfaces (BMIs), translate brain activity into commands to control external devices, such as computers, wheelchairs, or assistive robots^1^. BCIs can detect human intention in the absence of physical inputs so they can be used to assist people with movement disorders and provide an additional communication channel between humans and machines^2^.

Among the modalities that can be captured and decoded from the brain, the Steady-State Visual Evoked Potential (SSVEP) is one of the most widely used as it can be captured non-invasively using electroencephalography (EEG) with relatively high signal-to-noise ratio and requires minimal user training^3^. The SSVEP is an automatic response of the visual cortex in reaction to periodic visual stimulation^4,5^. The SSVEP responses show the same frequencies as the stimulation frequencies as well as the harmonics of the frequencies^4,6^. The existence of harmonics enables higher SSVEP classification accuracy^7^ but, at the same time, limits the selection of frequencies when constructing SSVEP-based BCIs^8^. Human brains respond to a constrained range of frequencies with optimal range identified as 12–18 Hz^4,9,10^ so, in cases where a large number of commands to be shown at once (i.e., a large number of frequencies need to be selected for stimulation), the frequencies become very close to each other and this makes decoding a very challenging task.

To increase the capacity of SSVEP-based BCIs to produce large numbers of commands, multi-frequency SSVEP was first proposed in 2010^11^ and different multi-frequency stimulation methods have since been developed^12–16^. The multi-frequency stimulation methods combine multiple frequencies in each stimulus. Therefore, by using different combinations of input frequencies, more stimuli can be represented with a smaller number of input frequencies. This makes multi-frequency SSVEP superior to single-frequency SSVEP when the number of targets becomes large because multi-frequency SSVEP does not require as many frequencies as single-frequency SSVEP, so a larger frequency interval can be maintained^17^. However, it was not until 2020 that studies on understanding better frequency selection in multi-frequency SSVEP were conducted^18,19^ and, in 2021, the first training-free decoding algorithm for multi-frequency SSVEP was developed^20^. It was demonstrated that multi-frequency SSVEP has more complex frequency components compared to traditional single-frequency SSVEP, including the existence of interactions between the input frequencies^12–15,20^. This feature creates redundancy in the information carried by the signal that can be used in decoding. Even though multi-frequency SSVEP has demonstrated its potential in delivering large numbers of commands, the research in this field is still lagging behind that of single-frequency SSVEP.

One way to facilitate research and encourage more people to study a topic is to create relevant datasets and make them widely accessible. The benchmark dataset^21^ for SSVEP-based BCIs has been used in over 200 scholarly works (based on Google Scholar citations) since its publication in 2017. Following this, more datasets on SSVEP have been published that focus on collecting SSVEP across multiple days and in multiple frequency bands^22^, SSVEP collected in a closer-to-real-world application setting^23^, comparing SSVEPs collected with wet and dry EEG electrodes^24^, SSVEP in the ageing population^25^, and feature-based selective attention in SSVEP^26^. BCI datasets that include SSVEP components were assembled to investigate BCI illiteracy^27^, facilitate the development of BCIs when users are in mobile situations^28^, and hybrid BCI combining EEG and other biosignals^29^. However, all of these datasets use single-frequency SSVEP, and there is not yet a publicly available dataset for multi-frequency SSVEP.

In this work, we constructed the first Open Access dataset for multi-frequency SSVEP^30^. Our dataset includes SSVEP collected from 35 participants with dry EEG electrodes. All participants were presented with single-frequency, dual-frequency, and tri-frequency visual stimulation using up to three different stimulation methods for each modality. EEG data is provided in complete session format to allow everyone to have access to all details in the recordings and thus simulate a real-time experiment experience. Matlab scripts are included to assist in separating data into trial lengths.

## Methods

### Participants

Thirty-five volunteers (aged 19 to 42 years, 25.91 mean ±5.30 standard deviation) participated in this experiment, who are free of neurological or facial muscle conditions. Out of the 35 participants, 25 were naïve to BCIs (had never previously participated in a BCI experiment); 27 were naïve to SSVEP-based BCIs (had never previously participated in an SSVEP experiment). For the eight experienced SSVEP-based BCI participants, the time since they last participated in an SSVEP experiment ranged from 4 months to 36 months (11.36 ± 10.38 months, mean ± standard deviation). Two participants were left handed and the rest were right handed. All participants had normal or corrected-to-normal vision (e.g., with glasses or contact lenses).

This study was approved by the University of Melbourne Human Research Ethics Committee (Project ID 24178). Written consent was collected from each participant. Each participant was compensated with an AUD $20 gift card.

### EEG Setup

EEG was recorded with g.USBamp and g.SAHARA dry electrodes (g.tec medical engineering GmbH, Austria) inside a Faraday shielded room. Brain activities were measured from six channels, PO3, POz, PO4, O1, Oz, and O2, according to the international 10-10 system. Reference and ground electrodes were positioned on left and right mastoids, respectively. Dry electrodes were selected due to the ease of setting up without gelling, which makes it more convenient in real-world applications.

During data acquisition, a 0.5–100 Hz band-pass filter and a 50 Hz notch filter were applied to all channels in g.USBamp settings. Data was recorded at a sampling rate of 512 Hz.

### Stimulation setup

An Alienware monitor AW2518HF (24.5 inch, 1920 × 1080, DELL Technologies, USA) was used to present all visual stimulation in this study. Participants sat in a chair at a distance of 70 cm from the screen measured from their eyes and with their head centred to the screen.

Stimulation was delivered through an interface programmed in Unity (Unity Technologies, USA) that ran on an EliteBook 840 G5 laptop (Hewlett-Packard, USA) with Core i7-8550U CPU @ 1.80 GHz, 16 GB RAM (Intel, USA) and UHD Graphics 620 integrated graphics unit (Intel, USA). The programmed interface displayed stimuli (targets) in white squares of size 108 × 108 pixels on a black background and 108 pixels gaps between adjacent targets in both vertical and horizontal directions. The interface was set to a 120 Hz refresh rate.

The frequencies used in this study were 7, 11, 13, 17, 19, and 23 Hz. These are prime numbers that are in the most responsive range of SSVEP^4^. The combinations of these six frequencies made six single-frequency targets, 15 dual-frequency targets (${C}_{2}^{6}=15$), and 20 tri-frequency targets (${C}_{3}^{6}=20$). Table 1 lists the frequencies and frequency combinations used in single-, dual-, and tri-frequency stimulation.

**Table 1:** Frequencies (in Hz) used in each target in single-, dual-, and tri-frequency stimulation.

Target #	Frequencies (Hz)		
	Single Frequency	Dual Frequency	Tri Frequency
1	7	7, 11	7, 11, 13
2	11	7, 13	7, 11, 17
3	13	7, 17	7, 11, 19
4	17	7, 19	7, 11, 23
5	19	7, 23	7, 13, 17
6	23	11, 13	7, 13, 19
7	—	11, 17	7, 13, 23
8	—	11, 19	7, 17, 19
9	—	11, 23	7, 17, 23
10	—	13, 17	7, 19, 23
11	—	13, 19	11, 13, 17
12	—	13, 23	11, 13, 19
13	—	17, 19	11, 13, 23
14	—	17, 23	11, 17, 19
15	—	19, 23	11, 17, 23
16	—	—	11, 19, 23
17	—	—	13, 17, 19
18	—	—	13, 17, 23
19	—	—	13, 19, 23
20	—	—	17, 19, 23

**Fig. 1:**
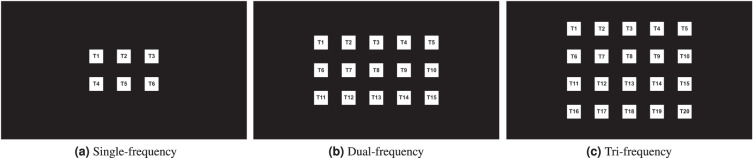
Stimuli layouts in single-frequency, dual-frequency, and tri-frequency tests. The 6 selected frequencies make 6 targets (2 × 3) in single-frequency, 15 (3 × 5) in dual-frequency, and 20 (4 × 5) in tri-frequency tests. “T” for target.

**Fig. 2:**
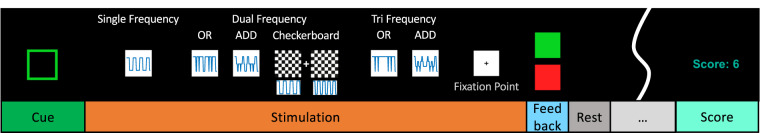
Trial structure and visual representations of the stimulation methods. Each trial starts with a 1 s cue (green frame around the intended target), followed by 5 s stimulation with a fixation point shown at the centre of the target. The collected SSVEP was online decoded and outcome fed back to the participant for 0.5 s right after stimulation finished with the target turning to solid green or red indicating correct or incorrect decoding output, respectively. A 0.5 s rest period was provided at the end of each trial. Note that in tri-frequency tests, Feedback and Rest were swapped to allow sufficient time for decoding. By the end of the test, a score is shown on the screen indicating the number of correctly decoded trials.

The stimuli layouts in single-, dual-, and tri-frequency tests are shown in Fig. 1. Simple flicking was used in delivering single-frequency stimulation. Three different stimulation methods were used in dual-frequency stimulation and two were used in tri-frequency stimulation. Details will be explained below; visual representations of the stimulation methods can be found in Fig. 2.

#### Single-frequency stimulation

Single-frequency stimulation was delivered as square waves flickering at full brightness. The six targets were laid out in a 2 × 3 matrix, as shown in Fig. 1a. The signal for each frequency was generated using

1 $${\rm{u}}=\frac{1}{2}{\rm{sgn}}(\sin (2\pi ft))+\frac{1}{2},$$

where *f* is the stimulation frequency and sgn() is the sign function.

#### Dual-frequency stimulation

Three different methods were used in dual-frequency stimulation: two frequency superposition methods (OR and ADD)^15^ and checkerboard^12^.

In dual-frequency superposition, two square waves are superimposed,

2 $${{\rm{S}}}_{{\rm{OR,2}}}={{\rm{u}}}_{{\rm{1}}}\vee {{\rm{u}}}_{{\rm{2}}}$$

3 $${{\rm{S}}}_{{\rm{ADD,2}}}=\frac{1}{2}{{\rm{u}}}_{{\rm{1}}}+\frac{1}{2}{{\rm{u}}}_{{\rm{2}}},$$

where u_1_ and u_2_ are signals generated from Eq. (1) using two different frequencies *f*_1_ and *f*_2_ In frequency superposition OR with two stimulation frequencies (S_OR, 2_), the OR logic is applied to the two square waves as shown in Eq. (2), where the stimulation is ON (1) when either (or both) of the signals is ON, and OFF (0) when both of the signals are OFF. Frequency superposition ADD with two frequencies (S_ADD, 2_) is achieved by reducing the brightness of each signal by half, then summing the brightness from the two signals, as described in Eq. (3).

The checkerboard method delivers the two stimulation signals separately, with its two patterns represented in the alternating squares. In this study, 8-by-8 checkerboards are used in place of each solid square stimulus.

The fifteen dual-frequency targets were shown in a 3 × 5 layout (Fig. 1b).

#### Tri-frequency stimulation

Similar to dual-frequency stimulation, frequency superposition OR and ADD were used in presenting tri-frequency stimulation. However, the checkerboard method was excluded as it does not support more than two frequencies shown at a time.

4 $${{\rm{S}}}_{{\rm{OR,3}}}={{\rm{u}}}_{{\rm{1}}}\vee {{\rm{u}}}_{{\rm{2}}}\vee {{\rm{u}}}_{{\rm{3}}}$$

5 $${{\rm{S}}}_{{\rm{ADD,}}3}=\frac{1}{3}{{\rm{u}}}_{1}+\frac{1}{3}{{\rm{u}}}_{2}+\frac{1}{3}{{\rm{u}}}_{3},$$

where u_3_ is the signal generated with a third frequency. In tri-frequency stimulation with frequency superposition, the formulations are similar to those in dual-frequency stimulation. In OR (S_OR, 3_), instead of two signals, we now add a third signal u_3_ as shown in Eq. (4). In ADD (S_ADD, 3_), the brightness of each signal is reduced to one third, as shown in Eq. (5).

The twenty tri-frequency targets were laid out in a 4 × 5 grid (Fig. 1c).

### Experimental protocol

#### Experiment structure

**Fig. 3:**
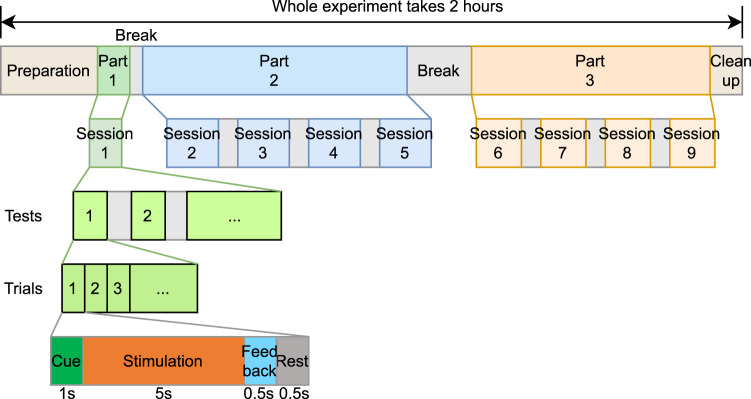
Experiment Structure. The whole experiment takes 2 hours to complete. The three parts of the experiment each focus on single-frequency, dual-frequency, and tri-frequency setups, respectively. Sessions have different session lengths and different numbers of tests. Details on sessions can be found in Fig. 4. Tests have 6 trials in part 1, 15 trials in part 2, and 20 trials in part 3, consistent with the numbers of targets in single-, dual-, and tri-frequency setups.

The experiment consisted of nine sessions, with session 1 testing single-frequency stimulation, sessions 2–5 testing dual-frequency, and sessions 6–9 testing tri-frequency. Three-minute breaks were provided between the sessions. A 10 minute break was placed between sessions 5 and 6 when the participant had finished all single- and dual-frequency sessions and before they started tri-frequency sessions. All breaks were adjusted to the participant’s need to minimise fatigue. Figure 3 depicts the structure of the experiment. The whole experiment required 2 hours to complete including the preparation and clean-up time (dry electrodes were used so experimenter only need to remove the cap from the participant during the clean-up). The experiment was completed in one sitting.

**Fig. 4:**
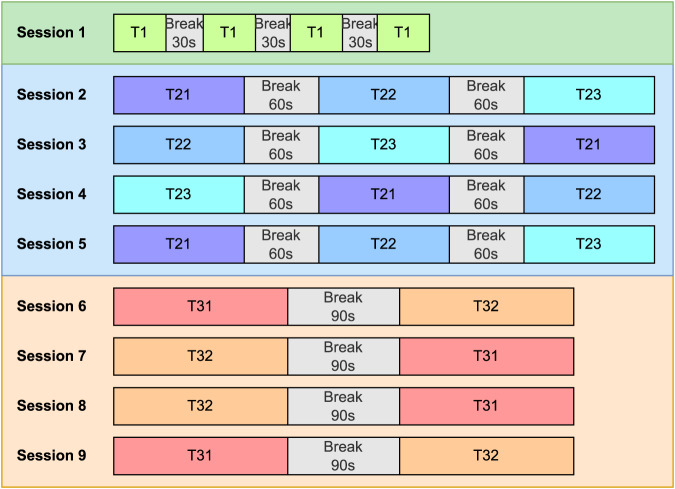
Session structure in the experiment. Test sequences for participant 1 are shown as an example. Session 1 includes the 4 repeated tests on single-frequency (T1). Sessions 2–5 are the 4 repeats of the three dual-frequency tests (T21, T22, and T23) with the test sequence in each session shuffled according to Table 2. Sessions 6–9 repeat the two tri-frequency tests (T31 and T32) 4 times in an AB-BA-BA-AB format alternated with BA-AB-AB-BA between the participants.

Each setup was tested four times. In session 1, the single-frequency setup (T1) was tested four times in a row. In sessions 2–5, the three dual-frequency setups (T21: frequency superposition OR; T22: frequency superposition ADD; T23: checkerboard) were tested once in each session. Therefore, each session included three tests in a balanced randomised sequence. Table 2 lists all sequences used in the experiment and the participants that used each sequence. Sessions 6–9 tested the two tri-frequency setups (T31: frequency superposition OR; T32: frequency superposition ADD) with each session running each setup once. The tri-frequency sessions followed an AB-BA-BA-AB sequence alternating with BA-AB-AB-BA between the participants (participants with odd indices followed AB-BA-BA-AB, even indices followed BA-AB-AB-BA). Figure 4 shows the structure of each session. Test sequences for participant 1 are labelled in this figure as an example.

**Table 2:** Sequences for dual-frequency sessions (sessions 2–5) and the list of participants that used each dual-frequency sequence.

Seq.#	Session 2	Session 3	Session 4	Session 5	Participants #
1	T21, T22, T23	T22, T23, T21	T23, T21, T22	T21, T22, T23	1, 10, 19, 28
2	T23, T21, T22	T22, T23, T21	T21, T23, T22	T22, T21, T23	2, 11, 20, 29
3	T23, T22, T21	T21, T23, T22	T23, T22, T21	T22, T21, T23	3, 12, 21, 30
4	T22, T21, T23	T21, T23, T22	T23, T22, T21	T22, T21, T23	4, 13, 22, 31
5	T23, T22, T21	T21, T23, T22	T22, T23, T21	T21, T22, T23	5, 14, 23, 32
6	T23, T21, T22	T22, T23, T21	T23, T21, T22	T21, T22, T23	6, 15, 24, 33
7	T23, T21, T22	T21, T22, T23	T22, T23, T21	T23, T21, T22	7, 16, 25, 34
8	T22, T23, T21	T21, T22, T23	T23, T22, T21	T21, T23, T22	8, 17, 26, 35
9	T22, T21, T23	T23, T22, T21	T22, T21, T23	T21, T23, T22	9, 18, 27

#### Trial structure

Trials were the smallest components in this experiment. Each trial started with a 1 s cue (green frame) to show the participant which target they should attend to. This was followed by a 5 s stimulation period with a fixation point provided to help them maintain attention on the target. Visual feedback (solid green or red block for correct or incorrect, respectively) was shown to the participant for 0.5 s after stimulation. Then the screen turned to solid black for 0.5 s as a resting period. Each trial was 7 s in total. Note that, in tri-frequency tests, the feedback and rest were swapped to allow sufficient time for the decoder to produce an output. Figure 2 shows the structure of the trials.

#### Test structure

A test refers to the action of going through all targets on the screen once each. In a single-frequency test (T1), one test has six trials as there are six targets. In a dual-frequency test (T21, T22, T23), one test has 15 trials. In a tri-frequency test (T31, T32), one test has 20 trials. In a test, participants went through the targets in a fixed sequence: from left to right and top to bottom. However, the stimuli were randomly shuffled on the screen to reduce undesirable bias. By the end of each test, a score is shown on the screen informing the participants of the number of correctly decoded trials, as shown in Fig. 2.

### Online decoding

Data were processed online with four training-free decoders operating in parallel to keep the experiment compact while minimising the effect of inaccurate modelling of each individual’s SSVEP responses in the decoding process. Canonical Correlation Analysis (CCA)^31^ for single frequency only, Multi-Frequency CCA (MFCCA)^20^ for multi frequency only, and Linear Diophantine Equation (LDE) decoding algorithms^32^ were used. The recorded EEG during the 5 s stimulation period were used in decoding.

#### CCA

Canonical Correlation Analysis (CCA)^31^ is a decoding algorithm that focuses on comparing the time-domain correlation *ρ* of the recorded multi-channel EEG **X** and predefined templates **Y** based on knowledge of the set of frequencies used. CCA looks for the weight vectors **W**_**X**_ and **W**_**Y**_, which constructs **x** = **X**^***T***^**W**_**X**_ and **y** = **Y**^***T***^**W**_**y**_, and maximises the correlation between **x** and **y,**

6 $$\mathop{max}\limits_{{{\bf{W}}}_{{\bf{X}}},{{\bf{W}}}_{{\bf{Y}}}}\rho ({\bf{x}},{\bf{y}})=\frac{E\left[{{\bf{x}}}^{T}{\bf{y}}\right]}{\sqrt{E\left[{{\bf{x}}}^{T}{\bf{x}}\right]E\left[{{\bf{y}}}^{T}{\bf{y}}\right]}}=\frac{E\left[{{\bf{W}}}_{{\bf{X}}}^{T}{{\bf{XY}}}^{T}{{\bf{W}}}_{{\bf{Y}}}\right]}{\sqrt{E\left[{{\bf{W}}}_{{\bf{X}}}^{T}{{\bf{XX}}}^{T}{{\bf{W}}}_{{\bf{X}}}\right]E\left[{{\bf{W}}}_{{\bf{Y}}}^{T}{{\bf{YY}}}^{T}{{\bf{W}}}_{{\bf{Y}}}\right]}},$$

where *E* is the mathematical expectation. The template **Y** in CCA is constructed with the sine and cosine signals at the stimulation frequency *f* and its harmonics,

7 $${{\bf{Y}}}_{CCA}(t)=\left[\begin{array}{c}\sin (2\pi ft)\\ \cos (2\pi ft)\\ \sin (2\pi 2ft)\\ \cos (2\pi 2ft)\\ \vdots \\ \sin (2\pi {N}_{h}\,ft)\\ \cos (2\pi {N}_{h}\,ft)\end{array}\right],$$

where *N*_*h*_ is the number of harmonics included in the formulation. For each stimulation frequency, a template **Y** is constructed and corresponding correlation calculated. The frequency that results in the highest correlation between **x** and **y** is selected as the decoder output.

In this work, two CCA configurations were used with *N*_*h*_ = 1 (decoder 1) and *N*_*h*_ = 2 (decoder 2).

#### MFCCA

Multi-Frequency Canonical Correlation Analysis (MFCCA)^20^ extends CCA to include the interactions between input frequencies into the template formulation. The templates **Y** in MFCCA are constructed with the sine and cosine signals at the stimulation frequencies and the integer linear combinations of the stimulation frequencies. Instead of bounding the size of **Y** with *N*_*h*_ as in CCA, it is bounded in MFCCA by order *N*_O_, defined as the sum of absolute values of the coefficients in the combination. For example, in dual-frequency SSVEP with stimulation frequencies *f*_1_ and *f*_2_, the linear integer combination of the two frequencies ${c}_{1}{f}_{1}+{c}_{2}{f}_{2}$, ${c}_{1},{c}_{2}\in {\mathbb{Z}}$ has order ${N}_{{\rm{O}}}=| {c}_{1}| +| {c}_{2}|$.

An example of the template formulation with two input frequencies up to order 2 is

8 $${{\bf{Y}}}_{\mathrm{MFCCA},{N}_{{\rm{O}}}=2}(t)=\left[\begin{array}{c}\sin (2\pi {f}_{1}t)\\ \cos (2\pi {f}_{1}t)\\ \sin (2\pi {f}_{2}t)\\ \cos (2\pi {f}_{2}t)\\ \sin (2\pi (2{f}_{1})t)\\ \cos (2\pi (2{f}_{1})t)\\ \sin (2\pi (2{f}_{2})t)\\ \cos (2\pi (2{f}_{2})t)\\ \sin (2\pi ({f}_{1}+{f}_{2})t)\\ \cos (2\pi ({f}_{1}+{f}_{2})t)\\ \sin (2\pi |{f}_{1}-{f}_{2}|t)\\ \cos (2\pi |{f}_{1}-{f}_{2}|t)\end{array}\right].$$

The two configurations selected for MFCCA in this work are *N*_O_ = 1 and *N*_O_ = 2 (the best performing settings as identified by Mu and colleagues^32^).

#### LDE

The Linear Diophantine Equation (LDE) decoder^32^ is capable of decoding both single-frequency and multi-frequency SSVEP. In LDE, the top *N*_*p*_ frequency peaks in the recorded SSVEP are first identified, then the coefficient(s) of the identified peak frequency in relation to the input stimulation frequency/frequencies are calculated through solving the formulated LDE. The frequency/frequency pair that has the highest number of integer solutions in solving the LDEs and lowest sum of orders is regarded as the decoder output.

The two LDE configurations for both single-frequency and multi-frequency decoding are selected as *N*_*p*_ = 9, *N*_O_ = 4 (the best performing setting as identified by^32^) and *N*_*p*_ = 12, *N*_O_ = 2 (decoders 3 and 4, respectively).

## Data Records

The dataset^30^ can be accessed on Figshare from 10.26188/22015694.

The dataset includes raw EEG data collected from 35 participants accompanied by metadata containing non-identifiable details of the participants. Data available in both.mat and.csv format. Matlab scripts are provided to assist users in preparing the data (.mat) in a more accessible form.

All nine sessions of EEG data from all participants are included in the dataset. Sessions are in data files named “P##_Ses#” where ## is a two-digit index for the participant, e.g. “P01”, and the last # is the session number (1–9). All data in .csv format are included in data_in_csv.zip.

### EEG Data

EEG data were recorded in Simulink and Matlab 2015a (MathWorks Inc., USA). In all recordings, the data has 10 rows. The first row records timestamps, rows 2 to 7 are the six EEG channels (PO3, POz, PO4, O1, Oz, O2, respectively), row 8 contains triggers, row 9 is processed from row 8 to show stimulation periods, and row 10 has decoder outputs in the online experiment.

The trigger signal in row 8 labels both onsets and offsets of the visual stimulation, where a positive integer labels the onset of each stimulation period and −1 labels the end of stimulation in each trial. The value of each onset trigger is the frequency index in each trial: 1–6 in single-frequency, 1–15 in dual-frequency, and 1–20 in tri-frequency. The decoder output in row 10 is an 8-digit integer (sometimes appears as 7-digit as the ‘0’ on the first digit is omitted) where the first two digits are the index (01–20) of the target decoded by decoder 1; the third and forth digits are the index of the target decoded by decoder 2, etc.

The script dataset_processData.m extracts the data (.mat) in sessions to data in trials based on the trigger information. The extracted data will be stored in a separate folder, still keeping one participant for each folder. Trials are named “P##_T#_R#_#”, where T# is the test name (e.g. T21), R# is the number of the repetition of the test (R1-R4), and the last # is the trial number/frequency index, which is equal to the trigger value.

### Metadata

Metadata dataset_metadata.xlsx includes non-identifiable participant information: sex and gender, age, dominant hand, and whether they have previous experience with EEG-based BCI and SSVEP-based BCI.

## Technical Validation

The data quality was validated through inspection of both time domain and frequency domain signal profiles, order distribution in multi-frequency tests, signal-to-noise ratios, and decoding accuracies.

### Signal profile in time and frequency domains

**Fig. 5:**
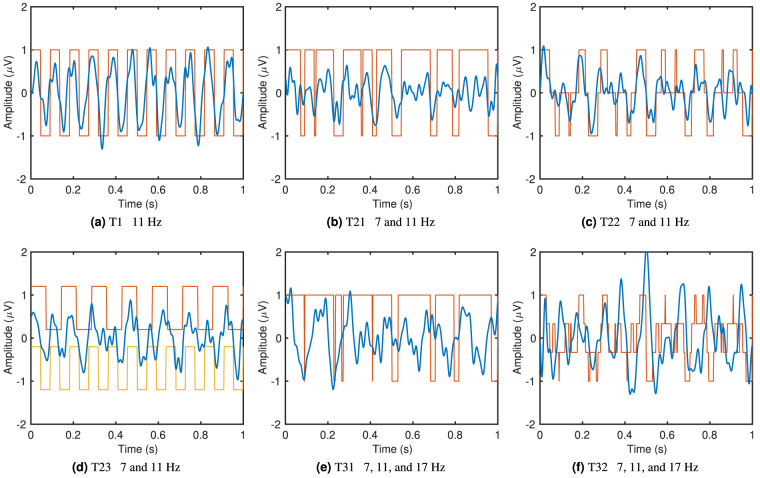
Examples of time domain waveforms from the six tests. (**a**) T1 11 Hz, (**b**) T21 7 and 11 Hz, (**c**) T22 7 and 11 Hz, (**d**) T23 7 and 11 Hz, (**e**) T31 7, 11, and 17 Hz, (**f**) T32 7, 11, and 17 Hz. Orange and yellow (only in (**d**)) show the waveforms of the stimulation signals. Blue plots the average SSVEP of all participants.

Figure 5 shows examples of the averaged time domain waveforms of the recorded SSVEPs overlaid on the waveforms of the stimulation signals. Here, 11 Hz in the single-frequency test T1 (Fig. 5a), 7 and 11 Hz in dual-frequency tests T21, T22, and T23 (Fig. 5b,d), and 7, 11, and 17 Hz in tri-frequency tests T31 and T32 (Fig. 5e,f) are shown as examples. The averaged waveforms were obtained by averaging the SSVEP from all participants in all four repeats, then band-pass filtering between 5 and 45 Hz, and finally cutting the 5 s data into five 1 s epochs with no overlap and averaging across all.

In single-frequency SSVEP, the waveform matches the stimulation signal very well, with harmonics visible. Dual-frequency and tri-frequency SSVEP waveforms still follow the corresponding stimulation signals in general, but the patterns are less prominent due to the additional stimulation frequencies and the complex interactions between them. It can also be observed from the plots that different stimulation methods trigger different SSVEP responses. The responses from frequency superposition ADD and the checkerboard pattern seem to follow the stimulation signal closer than the responses from frequency superposition OR.

**Fig. 6:**
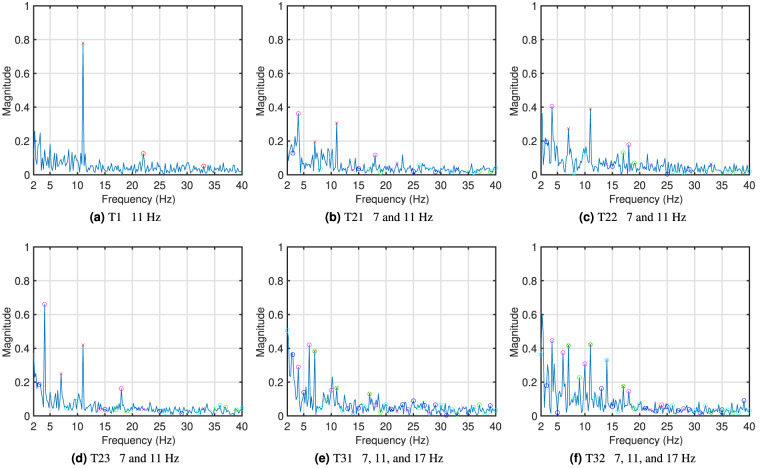
Examples of frequency domain magnitudes from the six tests. (**a**) T1 11 Hz, (**b**) T21 7 and 11 Hz, (**c**) T22 7 and 11 Hz, (**d**) T23 7 and 11 Hz, (**e**) T31 7, 11, and 17 Hz, (**f**) T32 7, 11, and 17 Hz. Red crosses label stimulation frequencies. In (**a**), red circles label harmonics. In (**b**–**f**), crosses label harmonics of the stimulation frequencies and circles label linear integer combinations of the stimulation frequencies. Different colours represent different numbers of harmonics or orders of interaction: magenta: 2, blue: 3, cyan: 4, green: 5.

Figure 6 shows the frequency domain components in the recorded SSVEPs. It is expected that clear peaks should be observed at the stimulation frequencies as well as their harmonics in single-frequency SSVEP^4^, and harmonics and integer linear combinations between the stimulation frequencies in multi-frequency SSVEP^15^. We can see by the markers labelling input frequencies and their harmonics and interactions that the expected frequency domain features are clearly visible for all stimulation types.

**Fig. 7:**
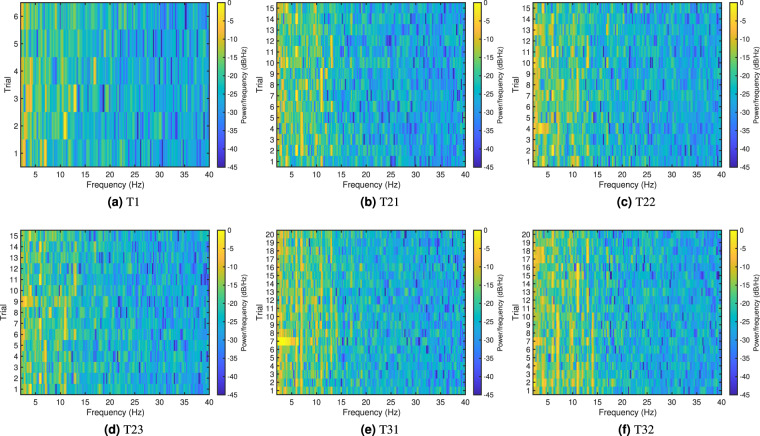
Estimated power spectral density of the average SSVEP of all participants in each trial in tests (**a**) T1, (**b**) T21, (**c**) T22, (**d**) T23, (**e**) T31, (**f**) T32. The trial number is the same as target index. Stimulation frequency in each target can be found in Table 1.

The frequency domain characteristics are further shown as estimated power spectral density (PSD) in Fig. 7. The PSDs were calculated using the short-time Fourier transform with the 5 s SSVEP recordings averaged across all participants in all repetitions. Each subplot in Fig. 7 shows the PSDs of each trial, or target, in each test. The stimulation frequencies of each trial/target in each test can be found in Table 1. From the figure, except the highlights on the stimulation frequencies and their harmonics and interactions, we can see that the power distribution across the spectrum is relatively consistent with slightly higher power in alpha and low-beta ranges as the number of stimulation frequency increases. This is partially due to the more complex frequency characteristics in multi-frequency SSVEPs where the integer linear combinations of the input frequencies can also be found in the recorded SSVEP.

From the above observations, we can conclude that the recorded EEG have the expected SSVEP responses.

### Order profile

**Fig. 8:**
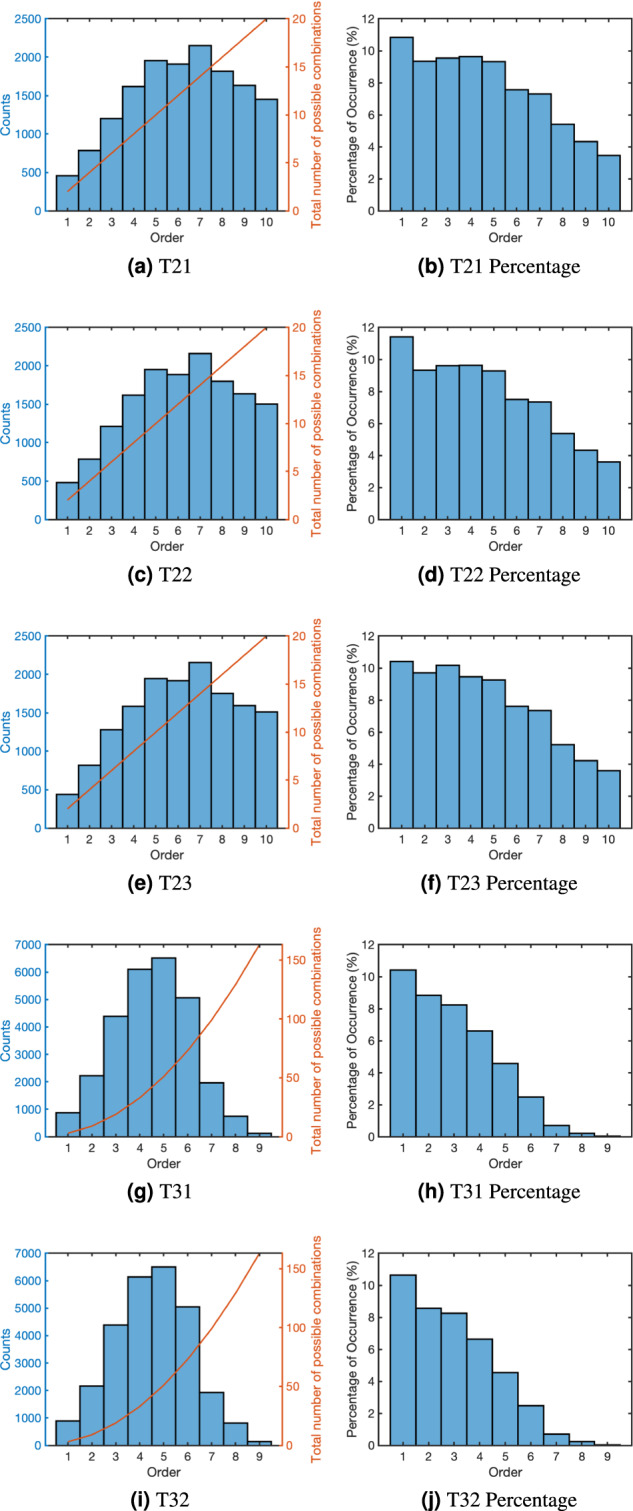
Histograms of orders of the top 10 peaks in each test. Figures on the left show the total number of times each order was observed in the top 10 peaks from each trial and the total number of possible combinations that can be made by the input frequencies at each order. Figures on the right show the percentage of all possible combinations that were found in the top peaks.

One important feature in multi-frequency SSVEP is the order of the interactions, which is defined as the sum of absolute values of the coefficients of interactions^20^. Fig. 8 shows the distribution of orders in the top 10 peaks in each trial in the five multi-frequency tests. Plots on the left hand side show the number of times each order was observed in the top 10 peaks (left/blue axis) and the total number of possible combinations (right/red axis). The plots on the right then show the percentage of the combinations observed out of all possibilities at each order using the information in the left hand side plots. The percentage of occurrence on the right agrees with the previous observations that harmonics and interactions at lower order have higher chance of being observed in the top peaks^20^.

The order distributions across the different stimulation methods only have slight variations when comparing among the same number of stimulation frequencies. However, the distributions in dual-frequency and tri-frequency show a clear difference. The decrease in the observed higher order peaks in tri-frequency may be attribute to the large number of overlapped frequencies in the harmonics and interactions, as can be seen in Fig. 6, and those peaks being identified and labelled with a lower order and so excluded in the higher order bins.

### Signal-to-Noise Ratio (SNR)

Narrow-band and wide-band SNRs^23^ were calculated to further demonstrate signal quality. The narrow-band SNR is the ratio between the power at the stimulation frequencies and the sum of powers in the ten neighbours of the stimulation frequencies on the spectrum (five on each side). Wide-band SNR considers the whole spectrum by taking the ratio between the sum of powers at the stimulation frequencies along with their harmonics as well as interactions (in multi-frequency) and the sum of powers of the rest of the frequencies in the spectrum. The mathematical formulations of the SNRs (in dB) in *n* (narrow-band) or *w* (wide-band) and *SF* (single-frequency) or *MF* (multi-frequency) scenarios are provided below.

Single-frequency narrow-band SNR:

9 $${{\rm{SNR}}}_{{\rm{n,SF}}}=1{0\log }_{10}\frac{P(F)}{{\sum }_{k=1}^{5}\left[P\left(F-k\Delta f\right)+P\left(F+k\Delta f\right)\right]},$$

where *F* is the frequency of interest (stimulation frequency), *P* is the Power Spectral Density (PSD) of the signal, and Δ*f* is the frequency resolution of the PSD.

Single-frequency wide-band SNR:

10 $${{\rm{SNR}}}_{{\rm{w,SF}}}=1{0\log }_{10}\frac{{\sum }_{k=1}^{{N}_{h}}P(kF)}{{\sum }_{f=0}^{{f}_{s}/2}P(f)-{\sum }_{k=1}^{{N}_{h}}P(kF)},$$

where *N*_*h*_ is the number of harmonics to be considered, *f*_*s*_ is the sampling frequency, and *f*_*s*_/2 denotes the Nyquist frequency.

Multi-frequency narrow-band SNR:

11 $${{\rm{SNR}}}_{{\rm{n,MF}}}=1{0\log }_{10}\frac{{\sum }_{i=1}^{{N}_{f}}P({F}_{i})}{{\sum }_{i=1}^{{N}_{f}}{\sum }_{k=1}^{5}\left[P({F}_{i}-k\Delta f)+P({F}_{i}+k\Delta f)\right]},$$

where *N*_*f*_ is the number of frequencies in the stimulation (dual-frequency *N*_*f*_ = 2, tri-frequency *N*_*f*_ = 3) and *F*_*i*_ then denotes the *i*^*th*^ stimulation frequency.

Multi-frequency wide-band SNR:

12 $${{\rm{SNR}}}_{{\rm{MF}}}=1{0\log }_{10}\frac{{\sum }_{i=1}^{n}P({\mathscr{F}}(i))}{{\sum }_{f=0}^{{f}_{s}/2}P(f)-{\sum }_{i=1}^{n}P({\mathscr{F}}i)},$$

where ${\mathscr{F}}$ is the set of frequencies including the stimulation frequencies, their harmonics and integer linear combinations up to order *N*_O_, ${{\mathscr{F}}}_{{N}_{{\rm{O}}}}=\{{f}_{1},{f}_{2},\cdots \,,{f}_{n}\}$.

Before calculating SNRs, signals were filtered with a second-order Infinite Impulse Response (IIR) notch filter at 100 Hz with quality factor 35 to remove the harmonic of power line noise, and were averaged across all channels. All trials were considered in producing the SNR histograms.

**Fig. 9:**
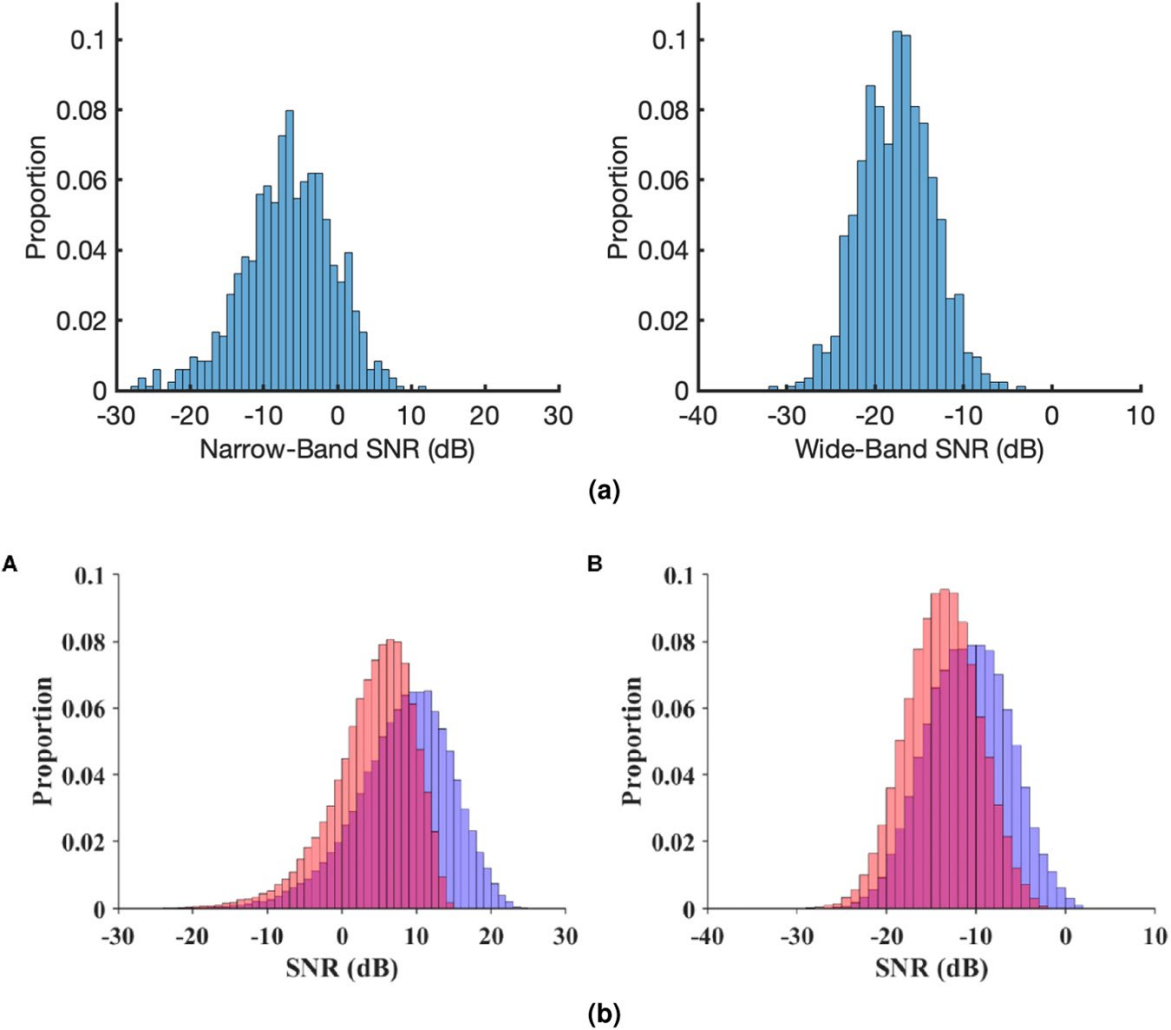
Narrow-band and wide-band signal-to-noise ratios (SNRs) from (**a**) T1 and (**b**) BETA (red) and Benchmark (purple) datasets (image directly taken from [23], used with permission). A: narrow-band SNR; B: wide-band SNR. Calculated with 5 s data bandpass filtered between 3 and 100 Hz.

We first compared the SNRs in T1 (single-frequency) with existing SSVEP datasets. To make the comparison as fair as possible, all trials in T1 were band-pass filtered under the same condition (between 3 and 100 Hz, using Matlab function “bandpass” with ‘ImpulseResponse’ set to ‘iir’, 0.85 ‘Steepness’, and 60 dB ‘StopbandAttenuation’), 5 s data were used in calculating the PSD to obtain a consistent 0.2 Hz frequency resolution, and number of harmonics *N*_*h*_ = 5. Figure 9 directly compares the narrow-band and wide-band SNRs in T1 and two publicly available SSVEP datasets: BETA dataset^23^ and Benchmark dataset^21^. It can be seen from the figures that the wide-band SNR in this study is similar to that in the BETA dataset, but the narrow-band SNR is around 10 dB lower than that in the two datasets. With the differences in setup taken into consideration, the results demonstrated a satisfactory quality of signals recorded in this dataset. There are some differences between the studies. First, in this work, a wider and, on average, higher frequency range was used compared to the other two studies, which may lower the SNR because SNR decreases as frequency increases^23^. Second, dry EEG electrodes were used in data collection compared to wet electrodes used in the two existing datasets. Dry electrodes are known to be more sensitive to artefacts and lead to lower decoding accuracy^24^; however, they are more practical with the simplified set up procedure (no gelling). Third, different recording devices, sampling rates, and channel selections were used in the studies.

**Fig. 10:**
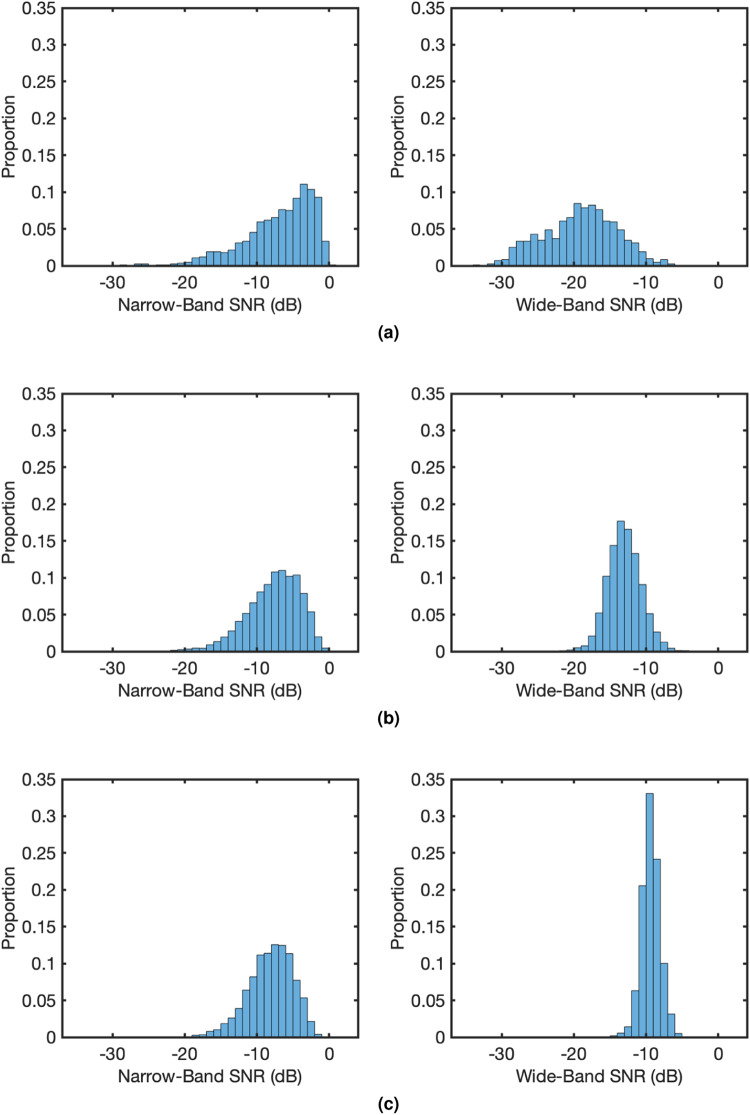
Narrow-band (left) and wide-band (right) signal-to-noise ratios for different numbers of stimulation frequencies: (**a**) single-frequency, (**b**) dual-frequency, (**c**) tri-frequency. Data bandpass filtered between 5 and 120 Hz and zero-padded to 10 s.

To examine the SNRs in multi-frequency SSVEPs, we compared the SNRs in single-frequency SSVEPs and dual- and tri-frequency SSVEPs. Different from above, we applied a band-pass filter between 5–120 Hz in Matlab to cover the 5^*th*^ harmonic of 23 Hz. Considering that complex interactions in multi-frequency SSVEP may result in adjacent integer frequencies both considered as signal, all trials were zero-padded to 10 s for a 0.1 Hz frequency resolution. This guarantees the 10 neighbours in narrow-band SNR calculation do not land on the signal frequencies. Figure 10 presents the distributions of narrow-band and wide-band SNRs in all single-, dual-, and tri-frequency trials. The histograms show the distributions of SNRs in all trials. The figures show a similar narrow-band SNR distribution in all cases with a small reduction in negative skewness as number of stimulation frequencies increases. In wide-band SNR, however, a clear reduction in variance with a positive shift in mean can be observed as number of stimulation frequencies increases. Overall, the SNRs fall within a reasonable range and demonstrated the quality of the signals in this dataset.

### Decoding accuracy

**Fig. 11:**
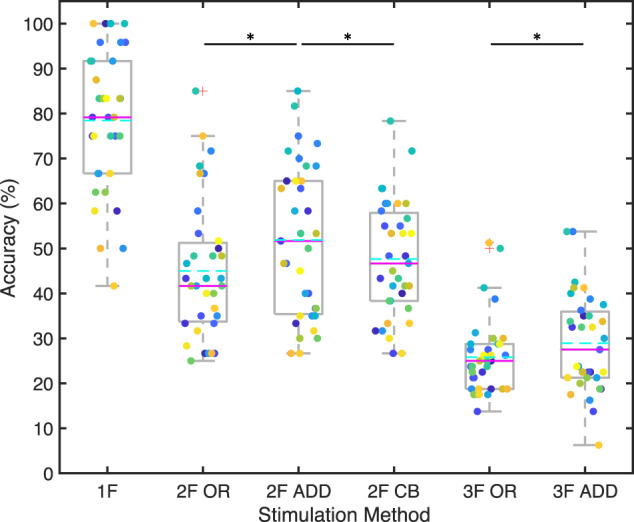
Accuracies of all participants in all tests. Results from each participant is a different colour. Solid magenta lines show median. Cyan dashed lines show mean. 1 F: single-frequency; 2 F: dual-frequency; 3 F: tri-frequency. OR: frequency superposition with OR; ADD: frequency superposition with ADD; CB: checkerboard. *significant difference (p < 0.05). Comparisons were only done between different stimulation methods on the same number of input frequencies (among the three 2 F groups, and between the two 3 F groups).

In addition to the analyses of the signal characteristics, decoding accuracies were also investigated. Figure 11 summarises the decoding accuracies from all participants in all tests. Each participant is labelled with a different colour. Boxes show 25–75 percentiles, whiskers show maximum and minimum values excluding outliers, red plus signs mark outliers that are more than 1.5 times the interquartile range (box size) away from the boxes, solid magenta lines label median values, and cyan dashed lines label mean values. Asterisks label statistical significance between the two groups at 5% level (*p* < 0.05). Comparisons were done only between different stimulation methods with the same number of input frequencies (i.e., 2 F OR vs. 2 F ADD, 2 F OR vs. 2 F CB, 2 F ADD vs. 2 F CB, 3 F OR vs. 3 F ADD) with the Wilcoxon signed rank test.

Detailed accuracies from each participant in each test are listed in Table 3. The listed accuracy from each participant was calculated as the average accuracy they achieved in the four repeats of each test based on the number of trials correctly identified in online decoding divided by the total number of trials.

**Table 3:** Accuracies of each participant in each test with mean and standard error of the mean (SEM) shown in the bottom of each column.

Participant #	Accuracy (%)					
	T1	T21	T22	T23	T31	T32
1	100.00	50.00	65.00	40.00	25.00	35.00
2	58.33	26.67	33.33	31.67	18.75	22.50
3	75.00	33.33	58.33	48.33	22.50	18.75
4	79.17	43.33	51.67	43.33	21.25	32.50
5	66.67	26.67	36.67	26.67	13.75	13.75
6	79.17	35.00	40.00	55.00	21.25	22.50
7	95.83	53.33	63.33	48.33	27.50	22.50
8	75.00	33.33	46.67	55.00	23.75	27.50
9	95.83	58.33	75.00	58.33	26.25	36.25
10	83.33	66.67	70.00	60.00	38.75	53.75
11	95.83	71.67	73.33	63.33	27.50	38.75
12	50.00	35.00	35.00	31.67	18.75	16.25
13	91.67	41.67	68.33	60.00	17.50	23.75
14	66.67	26.67	40.00	46.67	17.50	21.25
15	91.67	43.33	58.33	41.67	31.25	30.00
16	83.33	46.67	35.00	38.33	28.75	37.50
17	100.00	43.33	85.00	63.33	28.75	40.00
18	100.00	68.33	71.67	71.67	41.25	42.50
19	91.67	85.00	81.67	78.33	50.00	53.75
20	75.00	48.33	68.33	56.67	23.75	35.00
21	83.33	48.33	53.33	43.33	25.00	33.75
22	75.00	41.67	50.00	38.33	23.75	32.50
23	62.50	25.00	30.00	36.67	22.50	18.75
24	62.50	40.00	36.67	41.67	17.50	21.25
25	83.33	41.67	30.00	45.00	30.00	20.00
26	83.33	48.33	53.33	60.00	25.00	41.25
27	79.17	36.67	46.67	41.67	18.75	22.50
28	87.50	66.67	63.33	53.33	30.00	33.75
29	50.00	26.67	26.67	33.33	18.75	17.50
30	100.00	75.00	65.00	60.00	51.25	41.25
31	41.67	36.67	26.67	33.33	18.75	6.25
32	66.67	31.67	31.67	26.67	17.50	21.25
33	58.33	28.33	35.00	30.00	26.25	32.50
34	75.00	40.00	65.00	53.33	28.75	22.50
35	83.33	51.67	45.00	53.33	26.25	23.75
Mean	78.45	45.00	51.86	47.67	25.82	28.93
SEM	2.60	2.55	2.83	2.15	1.42	1.80

From Fig. 11 and Table 3, we can see that the average accuracies decrease as numbers of input frequencies increase. Overall, 85.7% (30/35) of participants achieved single-frequency accuracy over 60%, 48.6% (17/35) over 80%, 28.6% (10/35) over 90%, and 20% (5/35) over 95%. This is comparable to previous participant performances with dry electrodes^24^.

It is worth noting that the tasks are at similar levels of difficulties for the participants in terms of visually fixating on flickering blocks presented on the computer screen. A major contributor to the differences in decoding accuracies is the modelling accuracy of the multi-frequency SSVEP that was used in the decoding algorithms. This is also one of the reasons why we created this dataset. By making such a dataset public, we welcome others to join this research field to uncover the fundamentals in this complex response and improve its performance.

For future research, training-based decoding algorithms could also be explored to advance multi-frequency SSVEP, especially those that were shown to work well in single-frequency SSVEP decoding such as Task-Related Component Analysis (TRCA)^33^ and Task-Discriminant Component Analysis (TDCA)^34^.

## Usage Notes

The most straightforward way to use the data is to load it in Matlab (MathWorks Inc., USA) in .mat format. When working with data in .csv format, please keep in mind that these are large matrices that may need to be carefully taken care of in the file reading process.

The code dataset_processData.m provided with the dataset cuts data (.mat) into trials for easier access to the SSVEP recordings. Options are provided in the code to select participant(s) and session(s). A pdf version of the code is also included (dataset_processData.pdf).

Data may be used in part or in full session form to simulate an online BCI at 512 Hz sampling rate.

Metadata dataset_metadata.xlsx is also included to provide general non-identifiable participant information.

## Data Availability

Provided code can be found in the same repository as the dataset^30^, named dataset_processData.m. This code is written and tested in Matlab R2020a. No additional toolbox is required to run this code. At the top of the code, there are options to set folder name and path with variable folderName. Select participants and sessions of interest for processing (cut data from whole sessions into trials) with variables Participants and Sessions. A pdf version of the code is also included (dataset_processData.pdf).
